# Quantitative Assessment of Proliferative Effects of Oral Vanadium on Pancreatic Islet Volumes and Beta Cell Numbers of Diabetic Rats

**DOI:** 10.7508/ibj.2016.01.003

**Published:** 2016-01

**Authors:** Leila Pirmoradi, Ali Noorafshan, Akbar Safaee, Gholam Abbas Dehghani

**Affiliations:** 1 Dept. of Physiology, Shiraz University of Medical Sciences, Shiraz, Iran;; 2 Histomorphometry and Stereology Research Center, Shiraz University of Medical Sciences, Shiraz, Iran;; 3 Dept. of Pathology, Shiraz University of Medical Sciences, Shiraz, Iran;; 4 Endocrine and Metabolism Research Center**, **Namazi Hospital, Shiraz University of Medical Sciences, Shiraz, Iran

**Keywords:** Vanadium, Pancreas, Islet volumes, Rats

## Abstract

**Background:**

Oral vanadyl sulfate (vanadium) induces normoglycemia, proliferates beta cells and prevents pancreatic islet atrophy in streptozotocin-induced diabetic rats. Soteriological method is used to quantitate the proliferative effects of vanadium on beta-cell numbers and islet volumes of normal and diabetic rats.

**Methods:**

Adult male Sprague-Dawley rats were made diabetic with intravenous streptozotocin injection (40 mg/kg). Normal and diabetic rats were divided into four groups. While control normal and diabetic (CD) groups used water, vanadium-treated normal (VTN) and diabetic (VTD) groups used solutions containing vanadyl sulfate (0.5-1 mg/mL, VOSO_4_+5H_2_O). Tail blood samples were used to measure blood glucose (BG) and plasma insulin. Two months after treatment, rats were sacrificed, pancreata prepared, and stereology method was used to quantitatively evaluate total beta cell numbers (TBCN) and total islet volumes (TISVOL).

**Results:**

Normoglycemia persisted in VTN with significantly decreased plasma insulin (0.190.08 vs. 0.970.27 ng/dL, *P*<0.002). The respective high BG (53249 vs. 14446 mg/dL, *P*<0.0001) and reduced plasma insulin (0.260.15 vs. 0.540.19 ng/dL, *P*<0.002) seen in CD were reversed in VTD during vanadium treatment or withdrawal. While the induction of diabetes, compared to their control, significantly decreased TISVOL (1.90.2 vs. 3.030.6 mm^3^, *P*<0.003) and TBCN (0.990.1 vs. 3.20.2 x 10^6^, *P*<0.003), vanadium treatment significantly increased TISVOL (2.90.8 and 4.071.0 mm^3^, *P*<0.003) and TBCN (1.50.3 and 3.80.6 x 10^6^, *P*<0.03).

**Conclusion:**

Two-month oral vanadium therapy in STZ-diabetic rats ameliorated hyperglycemia by partially restoring plasma insulin. This action was through proliferative actions of vanadium in preventing islet atrophy by increasing beta-cell numbers.

## INTRODUCTION

Diabetes mellitus, a state of chronic hyper-glycemia, is characterized by increased insulin resistance of peripheral tissues (type 2 diabetes) or reduced pancreatic beta cell mass and absolute insulin storage (type 1 diabetes). At first, the increased insulin resistance is compensated by stimulation of pancreatic beta cells to raise insulin secretion^[^^1^^]^. Eventually, the persistent stimulation exhausts the pancreas, promotes apoptosis and reduces proliferation; all leading to beta cells exhaustion and degradation^[^^2^^]^.

Streptozocin (STZ), a toxic glucose analogue used in laboratory animals, irreversibly destroys pancreatic beta cells and induces type 1 diabetes^[^^3^^]^. Hyper-glycemia, low plasma insulin, atrophic pancreatic islets, reduced beta cell mass and depletion of islet insulin content are clear signs of STZ diabetes^[^^4^^]^. Studies carried out in the diabetic rats have demonstrated that oral vanadium dramatically improves peripheral tissue responsiveness to insulin and induces stable normoglycemia during treatment and after withdrawal^[^^5^^-^^8^^]^. Insulin immune-reactivity of isles of diabetic rats also has revealed that vanadium therapy prevents pancreatic islet atrophy, increases the reduced beta cell mass and subcellular organelles and islet insulin store^[^^7^^,^^9^^-^^11^^]^.

Vanadium might be a valuable supplement to insulin in the treatment of diabetes mellitus^[^^12^^]^. Clinical trials performed in diabetic patients have demonstrated that in type 1 diabetes, long-term oral vanadium consumption alongside with the reduction of high blood glucose lowers the required dose of daily insulin^[^^13^^]^. Decreased hepatic insulin resistance in type 2 diabetes consistently increased basal levels of insulin receptors and improved insulin signaling defect^[^^14^^]^.

The valuable insulinotropic effects of oral vanadium on the pancreatic beta cells of normal and STZ diabetes rats are well documented^[^^6^^,^^9^^,^^10^^]^. In spite of the presence of euglycemia in normal rats, vanadium proliferated beta cells and enlarged pancreatic islets^[^^7^^,^^9^^]^. Histological and ultrastructural studies of pancreas in diabetic rats also have demonstrated that oral vanadium consumption prevents pancreatic islet atrophy, renews the damaged beta cells and restores islet insulin store^[^^4^^,^^9^^,^^11^^,^^15^^-^^17^^]^. Though revalidating the insulinotropic effects of vanadium on pancreas, in the present study, we intended to quantitatively estimate the proliferative effects of vanadium on beta cells islets volume of normal and STZ-diabetic rats.

## MATERIALS AND METHODS

All protocols of the study were approved by the Institutional Animal Ethics Committee of the Shiraz Medical Sciences University (Shiraz, Iran), which follows NIH guidelines for care and use of animals (NIH publication No. 85-23, revised in 1996). Experiments were performed on male healthy Sprague Dawley rats (200-250 g). Animals were housed in standard cages in a room with controlled temperature (22-24°C), humidity (40-60%), and light period (07.00-19.00). Animals had free access to food (rat food, Parsdam, Tehran, Iran) and water *ad libitum*. 

**Fluid solutions**

The drinking fluid contained 3 g/L NaCl (in distilled water) to overcome the problems of natriuresis happening in diabetic rats^[^^6^^]^. Vanadium solution contained vanadyl sulfate (VOSO_4_ + 5H_2_O, Merck, Germany) in the drinking fluid at concentrations of 0.05 to 1 mg/mL. All solutions were freshly prepared every 3-5 days and stored in a dark cold room (4^o^C) until use.

**Blood samples**

The animals were slightly anesthetized with ether, and 500 µL blood was collected from the tip of snipped tail. Two µL blood was then used to measure BG with Gloucard-01 (Japan), and the rest was centrifuged (12,000 ×g) to separate the serum. Serum samples were then stored in a freezer (-70^o^C) for the assessment of plasma insulin.

**Routine measurements**

The drinking fluids were measured daily during the first two weeks of the study and then every other days. Body weight was determined every week. BG and plasma insulin was measured at times presented in the Results section.

**Induction of diabetes and maintenance of the animals**

Diabetes was induced with a single intravenous injection of freshly prepared streptozotocin solution (40 mg/kg in normal saline) through lateral tail vein, and the control normal (CN) animals received the same volume of normal saline^[^^7^^]^. Animals were housed in the same room but in separate cages (one per cage). Induction of diabetes was confirmed 48-72 h after STZ injection by the presence of hyperglycemia (BG = 350-400 mg/dL), polydipsia (fluid intake ≥100 mL/day), and polyuria (wet cage). 

**Experimental design and groups**

Animals were divided into four groups of six each. 1) CN group: 10 days after saline injection, the drinking water of control normal rats was switched to fluid solution and treatment continued for two months; 2) Control diabetic group (CD): similar to CN group, diabetic rats used fluid solution as drinking water for two months; 3) Vanadium-treated normal group (VTN): 10 days after normal saline injection, the drinking water was switched to vanadium solution (1 mg/ml VOSO_4_ + 5H_2_O in distilled water)[^7^^,^^11^]; 4) Vanadium-treated diabetic group (VTD): 10 days after STZ injection, the drinking water was replaced with vanadyl solution. In this group at the start of the experiment, the concentration of vanadyl was 0.05 mg/mL. With the reduction of fluid consumption (generally happened during the first two weeks of vanadium therapy), the concentration of vanadyl sulfate was gradually increased and treatment was continued at 1 mg/mL for two month^[^^11^^,^^18^^]^. 

**Estimation of pancreas volume**

At the end of two month experiments, animals were sacrificed under deep anesthesia (ketamine/xylazine 70/10 mg/kg). Pancreata gently were taken out, cleaned of fat and connective tissues and weighted. Scherle's immersion method was used to measure the primary volume (V_primary_) of the pancreas^[^^19^^,^^20^^]^. After tissue sectioning, processing, and staining of the sections, the area of the circular pieces was measured. The tissue sections stained with modified aldehyde fuchsin^[^^21^^]^. Then the isotropic, uniform random slabs of the pancreas were obtained with the orientator method^[^^20^^]^. Two circular pieces (2 mm diameter) were punched, and two causal slabs were obtained^[^^20^^]^. All the slabs and the circular pieces were embedded in a paraffin block. According to Bangle's method^[^^21^^]^, one 4-μm and one 20-μm sections were cut from the slabs and stained with modified Gomori's aldehyde fuchsin. The degree of atrophy d_(ath)_ was estimated using the following equation:

d_(ath)_ = 1 – (AA/AB)^1.5^

where AA and AB are the respective areas of each circular pieces of the pancreas after and before processing, sectioning and staining.

**Estimation of volume density of the pancreatic islets**

The microscopic slides were analyzed using a video microscope (MT-12, Heidenhain, Traunreut, Germany). The microscopic fields of each histological slide were sampled in a systematic random manner. Finally, the point-counting method was used at magnification of 140×. The following formula was applied to estimate the volume density of the islet (*V*_v_) on sections of 4-μm thickness^[^^20^^,^^22^^]^: 

V_v_ = *P*_(islet)_/*P*_(reference)_

where the respective *P*_ (islet)_ and *P*_(reference)_ are the numbers of the test points hitting the islet profile and the reference space. Then the following formula was applied to estimate the final islet volume “V_(islets)_”: 

V_(islets)_ = *V*_v_ × V_(primary)_ × [1 – d_(ath)_]

***where, V***_v_ ***is*** ***the volume density of islet*** ***s, and*** ***V***_(primary)_ ***is the primary volume of the pancreas. ***

*Numerical density of beta* *cells*

**Fig. 1:**
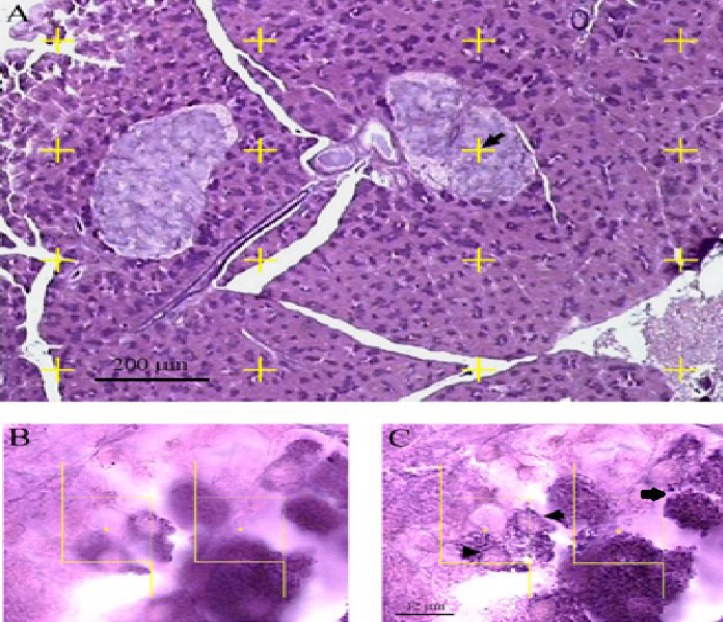
Optical scan for the estimation of the number of the beta cells. The cells that their nuclei did not appear in the beginning of the disector height (A) and appeared at the following optical scan of the disector height (B) were counted. The arrow in A indicates that one point from 16 points is landed on the island. B and C are optical disectors. Any nucleus (the arrow in disector height) that comes into focus was counted (C). The cells (the arrow heads) that hit the forbidden line of the frame were ignored (C

***According to the optical disector method*** ***,*** ***an oil immersion objective len*** ***s*** ***(100×, numerical aperture: 1.4, at final magnification of 3400×)*** ***,*** ***was used to count*** ***beta*** ***cells*** ^[^^20^^,^^22^^,^^23^^]^ ***. The subsequent formula was*** ***also*** ***used to estimate the numerical density (N***_V_ ***) sections of 20*** ***-*** ***μm thickness (*** Fig. 1 ***).***

**Table 1.** Water intake and body weight before treatment and at days 30 and 70 of the experiments

Groups (n = 6)		**Water consumption (mL/day)**				***Body weight (g)***		
		**Before**	**Day 30**	**Day 70**		**Before**	**Day 30**	**Day 70**
CN		33 ± 1	34 ± 1	35 ± 2		233 ± 11	265 ± 9	290 ± 9
CD		33 ± 2	202 ± 5	232 ± 15		213 ± 10	207 ± 12	203 ± 19
VTN		36 ± 1	25 ± 2	28 ± 5		237 ± 10	240 ± 9	230 ± 25
**VTD**		33 ± 1	40 ± 3	35 ± 4		232 ± 19	227 ± 22	219 ± 39
P *value*		0.6	0.001	0.001		0.6	0.06	0.001

Data are mean ± SD. CN, control normal; CD, control diabetic; VTN, vanadium-treated normal; VTD, vanadium-treated diabetic rats.

N_V_ = [ΣQ^-^/(h × a/f × Σp)] × (t/BA)

where, "a/f" is the area of the counting frame (here was 184 μm^2^), "h" is the height of the optical disector (here was 20 μm), "Σ*Q*^ –^" is the number of the beta cells counted in all the disectors, and "Σ*p*" is the total number of the counted frames, "BA" is the microtome block advance to cut the block (here was 20 μm), "t" is the mean of final section thickness (17.4 μm on the average)^[^^23^^]^.

To estimate the total number of the beta cells “N_(beta cell)_” the following formula was used:

N_(beta cell)_ = Nv x V_(islets)_

where V_(islets)_ is total islet volume.

**Statistical analysis**

Data are presented as mean ± SD. All values are presented as means ± SEM. Comparisons between the groups were performed by analysis of variance (ANOVA), followed by Tukey’s post-hoc test. *P*<0.05 was considered statistically significance. 

## RESULTS

**Body weight**

Changes in body weight are presented in Table 1. In CN rats, a steady-state rise in body weight was observed during two-month period, but no significant changes were observed in the body weight of VTN, CD, and VTD groups. 

**Daily water intake and vanadium consumption**

Table 1 presents changes in daily water consumption during two-month study. Daily water intake of normal rats of CN group was 33 ± 1 mL/day. Induction of diabetes significantly increased water intake in CD group. After one month, this increase reached to 202 ± 5 mL/day and after two months reached to 232 ± 15 mL/day. Water intake in VTN group was significantly decreased and finally reached to 28 ± 5 mL/day (*P*<0.001). Polydipsia seen in diabetic rats of CD group was slowly decreased in VTD. After one month, water consumption reached to 40 ± 3 mL/days *P*<0.001) and totally stabilized at 35 ± 5 mL/day. In this condition, the subsequent average vanadium consumption (as vanadyl sulfate) in VTN was 18.0 ± 3.2 mg/day and in VTD was 22.5 ± 2.5 mg/day. 

**Blood glucose and plasma insulin levels**

Fasting BG and plasma insulin levels (insulin) of normal and diabetic rats are presented in Table 2. Before the start of the experiments, the respective BG and insulin in CN were 87 ± 6 mg/dL and 1.0 ± 0.3 ng/dL, respectively. During two-month period, they did not significantly change (*P* = 0.6). Normoglycemia persisted in VTN at significantly low plasma insulin (*P*<0.002). Hyperglycemia observed in CD 10 days after STZ injection was worsened during two-month period (*P*<0.002). High blood glucose observed 10 days after STZ injection (or just before the start of vanadium treatment) was significantly decreased in VTD (*P*<0.001), and at the same time there was a significant increment in reduced level of plasma insulin (*P*<0.002). Nonetheless, in VTD, the increased level of plasma insulin was still statistically lower than CN group (*P*<0.005). 

**Fig. 2:**
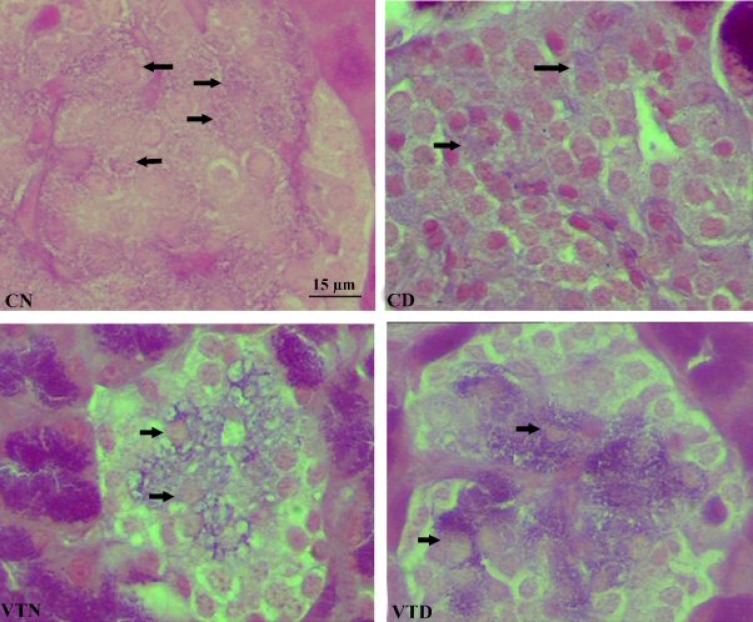
Modified Gomori's aldehyde-fuchsin staining of the pancreas. Control normal (CN), control diabetic (CD), vanadium-treated normal (VTN) and vanadium-treated diabetic (VTD) rats. Arrows show beta cells

Typical Gomori's aldehyde-fuchsin staining results of the pancreas obtained by histological examination are shown in Figure 2. The changes occurred in the islets of other groups were compared with pancreatic histology of the normal rats (Fig. 2, CN). Islets in CD were atrophied and beta cells were dispersed (Fig. 2, CD). In contrast to CN, islets of VTN group were larger in size, and more abundant beta cells situated centrally (Fig. 2, VTN). In VTD compared to CD, the damaged islets were partially repaired, and numerous normal beta cells were present in the center of the islets (Fig. 2, VTD). 

**Pancreatic islet volumes and total beta cell numbers**

Total pancreas weight and volume, total islet volumes and total beta cell numbers of untreated normal and diabetic rats as well as vanadium treated rats with the average body weight of 290 ± 9g are presented in Table 3. Induction of diabetes did not significantly change the weight and volume of the pancreas (910 ± 70 mg, *P* = 0.4), but there was a 63% reduction in the total islet volumes (840 ± 60 mm^3^, *P*<0.003) with 31% decreased total beta cell numbers (3.2 ± 0.2 × 10^6^, *P*<0.003). In VTN compared to CN, there were significant increases in the weight (1200 ± 190 mg, *P*<0.003) and volume (1100 ± 100 mm^3^, *P*<0.003) of the pancreas. The total islet volumes (4.07 ± 1.0mm^3^, *P*<0.003) and the total beta cell numbers (3.8 ± 0.6 × 10^6^, *P*<0.003) were also increased significantly. In VTD, compared to CD, without seeing noticeable changes in the weight (*P* = 0.5) or volume (*P* = 0.5) of the pancreas, clear decreases were observed in both total islets volume (15%, *P*<0.03) and total beta cell numbers (13%, *P*<0.03). 

**Table 2:** Blood glucose (mg/dL) and plasma insulin levels (μg/L) before treatment and day 70 of the experiments

**Groups** **(n = 6)**		**Blood glucose**			**Plasma insulin**			***P*** ** value**	
		**Before**	**Day 70**		**Before**	**Day 70**		**Glucose**	**Insulin**
CN		87 ± 6	90 ± 4		1.0 ± 0.3	0.97 ± 0.27		0.4200	0.150
CD		91 ± 7	532 ± 49		1.1 ± 0.2	0.26 ± 0.15		0.0001	0.002
VTN		92 ± 7	87 ± 5		0.9 ± 0.4	0.19 ± 0.08		0.0010	0.005
VTD		89 ± 7	144 ± 46		1.2 ± 0.4	0.54 ± 0.19		0.0400	0.004
*P *value		0.6	0.001		0.6	0.0001			

Data are mean ± SD. CN, control normal; CD, control diabetic; VTN, vanadium-treated normal; VTD, vanadium-treated diabetic rats.

## DISCUSSION

Earlier studies performed on laboratory animals have demonstrated that STZ, depending on its dose, extensively reduces beta cells mass and destroys pancreatic islet volume^[^^9^^,^^24^^]^. Additionally, the prolonged toxicity of hyperglycemia exhausts the remaining viable beta cells and worsens diabetes^[^^25^^]^. Studies performed on the diabetic rats showed that short-term vanadium treatment protects beta cell degeneration, and by increasing the reduced level of the plasma insulin induces normoglycemia during vanadium treatment and after withdrawal^[^^9^^,^^24^^]^. In the insulin-dependent diabetic rats, the combination of vanadium and insulin therapy (a minimum injection dose of insulin) and also one-year time are needed to extend normoglycemia after the withdrawal of insulin or vanadium^[^^18^^]^. Overall, regardless of the severity of diabetes, vanadium therapy needs a minimum level of plasma insulin, secreted endogenously, or received exogenously. This issue can assist vanadium to induce normoglycemia during treatment and after withdrawal^[^^7^^,^^9^^,^^18^^]^.

**Table 3:** Quantitative values of pancreas weight and volume, total islet volumes (TISVOL), and total beta cell numbers (beta cells) 70 days after the start of the experiments

**Groups** **(n = 6)**		**Weight (mg) **		**Volume (mm** ^3^ **) **			**Number (×10** ^6^ **)**
		**Pancreas**		**Pancreas**	**TISVOL**		**Beta cells**
CN		1020 ± 100		980 ± 100	3.03 ± 0.6		3.2 ± 0.2
CD		910 ± 70*		840 ± 60 *	1.9 ± 0.2 *		0.99 ± 0.1 *
VTN		1200 ± 190 *		1100 ± 100 *	4.07 ± 1.0 *		3.8 ± 0.6 *
VTD		950 ± 100		874 ± 90	2.9 ± 0.8**		1.5 ± 0.3 **

Data are mean ± SD. CN, control normal; CD, control diabetic; VTN, vanadium-treated normal; VTD, vanadium-treated diabetic rats.

* CD or VTN* vs.* CN group (*P*<0.003);

** VTD *vs.* CD (*P*<0.03)

The typical daily water intake, body growth weight, glycemic status, and pancreatic islet structures of the normal and diabetic rats presented here were used to compare the results of oral vanadium treatment on the normal and the diabetic rats. In this study high blood glucose (*P*<0.0001), polydipsia, polyuria (wet cage), and reduced plasma insulin (*P*<0.002) were clear signs of diabetes in the CD group. The existence of atrophic islets and reduced and dispersed beta cells indicated that the source of diabetes was the reduced plasma insulin due to islet atrophy, as well as the reduced islet insulin content and secretion^[^^6^^,^^7^^,^^9^^,^^16^^]^.

While normal rats of the CN group had a steady, positive growth of body weight, the induction of diabetes, after STZ injection or vanadium treatment reversed the growth of body weight, and at the end of the experiment, the body weight in the CD, VTN, and VTD groups was significantly lower than CN group (*P*<0.01). The suppression of the growth body weight seen in the CD group was conceivably linked with the impaired carbohydrate metabolism as a source of energy^[^^11^^,^^26^^,^^27^^]^. However, the suppressive action of vanadium on the appetite, via anorexigenic stimulation of the central nervous system, was possibly the cause of reduced growth body weight in the VTN or VTD group^[^^28^^]^. 

Studies have demonstrated that food restriction and weight loss may lower peripheral insulin resistance in type 2 diabetes and lowers the damaging effects of glucose toxicity on the pancreatic islets^[^^26^^,^^27^^]^. In contrast, the results of the current study and previous investigations indicated that the decreased growth body weight seen in VTD group was possibly due to food restriction^[^^7^^,^^16^^,^^28^^]^. However, the results of the present study precluded this possibility because the reduced body weight observed in the CD group was accompanied with worsened hyperglycemia. Therefore, we assume that in the VTD group, the insulin-mimetic actions of vanadium on the peripheral tissues could balance glucose metabolism and prevent the recurrence of diabetic symptoms. This fact is supported by other investigations indicated that the insulin-mimetic actions of vanadium reduces the insulin resistance of peripheral tissues, which improves glucose uptake and relieves hyperglycemia^[^^7^^-^^9^^,^^18^^,^^29^^,^^30^^]^. In a similar way, reports indicated that the insulin-mimetic action of vanadium in type 2 diabetic patients could improve carbohydrate metabolism and reverse hyper-glycemia^[^^4^^,^^5^^,^^9^^,^^18^^]^. 

Earlier reports have shown that the insulinotropic actions of vanadium can expand islet areas and increase beta cell mass in both normal and STZ diabetic rats^[^^7^^,^^18^^,^^31^^]^. The quantitative results of this study endorsed the fact that the expansion of islet areas seen in the VTN group was mainly due to the increased beta cell numbers but not the cell volume. In the diabetic rats of the CD group, although the real causes of islet atrophy was the reduced number of the beta cells, the diabetic rats in the VTD group inverted islet atrophy by repairing the injured beta cells. Furthermore the insulinotropic action of vanadium proliferated the beta cell and increased the islet volume^[^^4^^,^^7^^,^^9^^]^. 

The result of this study revealed that in the STZ diabetic rats, the main cause of the prolonged doiabeties was due to the reduced beta cell numbers. The insulinotropic actions of vanadium depressed beta cell death, and by the proliferation of the viable beta cells, it increased the insulin store of the pancreas and plasma insulin.
